# Cyclic Methacrylate Tetrahydropyrimidinones: Synthesis, Properties, (Co)Polymerization

**DOI:** 10.3390/polym14010107

**Published:** 2021-12-29

**Authors:** Victor A. Gerasin, Marina V. Zhurina, Natalia A. Kleshcheva, Nikolai A. Sivov, Dmitry I. Mendeleev

**Affiliations:** 1 A.V. Topchiev Institute of Petrochemical Synthesis (TIPS), Russian Academy of Sciences (RAS), Leninskii pr. 29, 119991 Moscow, Russia; gerasin@ips.ac.ru (V.A.G.); kleshcheva@ips.ac.ru (N.A.K.); sivov@ips.ac.ru (N.A.S.); 2 Winogradsky Institute of Microbiology, Research Center of Biotechnology, Russian Academy of Sciences, 119071 Moscow, Russia; mzhurik@gmail.com

**Keywords:** biocide, radical vinyl polymerization, polyguanidine

## Abstract

During radical polymerization of novel biocidal methacrylate guanidine monomers, a cyclic byproduct was discovered and identified as 2-imino-5-methyltetrahydropyrimidin-4(1H)-one (THP). Its methacrylate salt (MTHP) was synthesized and characterized via ^1^H and ^13^C NMR and pyrolysis chromatography. Synthesis conditions of both THP and MTHP were optimized to high yields, and both MTHP homopolymerization (in aqua) and copolymerization with diallyldimethylammonium chloride (in aqua in salt form) were successfully carried out with middle to high yields, providing a promising platform for potential tailored biocide polymers.

## 1. Introduction

The high-tech areas of modern technology are always dependent on progress in material science, and, as such, the importance of novel polymer and polymer materials development is paramount. Generally, the creation of a new polymer material consists of consecutive and independent stages of monomer synthesis, polymer synthesis, determining their properties and, at last, the preparation and testing of polymer material, with each stage being a specific field of study with different methods and particularities. Thus, the developments in polymer chemistry are usually focused on a single aspect, and in recent decades the trend of novel polymer development was skewed to extending the existing methods for large-scale polymer synthesis (chemical or physical modification of volume plastics, perfecting the catalytical systems, using the tried-and-proven monomers and methods for polymerization, etc.).

At this point, this classical approach is close to be exhausted, not being up to the challenge of creating the wide range of specialty materials for modern usage, and new polymer materials are to be employed, using principally novel methods and monomers in their synthesis and production. This especially concerns the essential field of biocidal polymers and polymer composites, able to be used as different kinds of materials—sanitary solutions, gels, powders, composites with volume-distributed active biocidal compounds for water treatment, composite materials, and protective coatings.

**Scheme 1:**
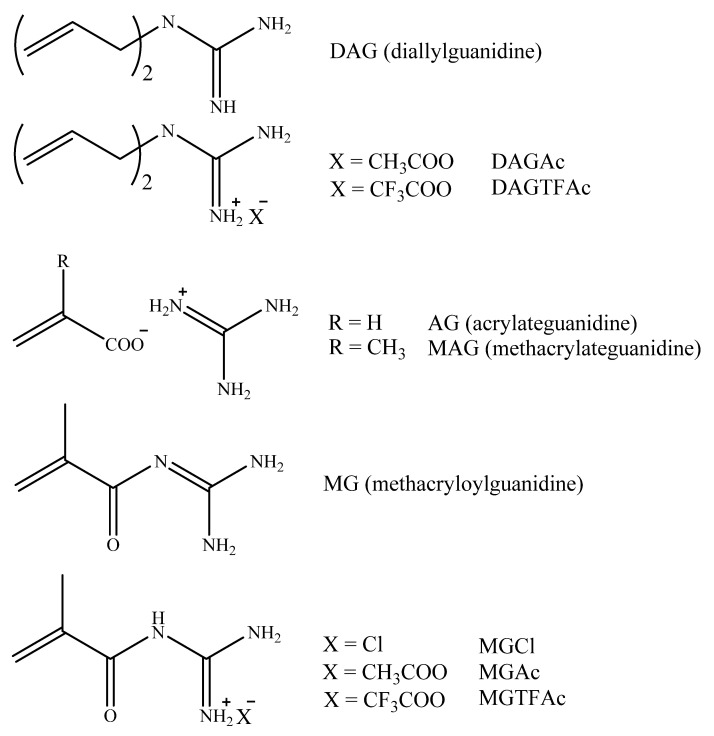
Guanidine-derived monomers.

We had pioneered techniques for synthesizing a class of novel guanidine-containing monomers [1] based on reactions of guanidine with (a) unsaturated or inorganic acids or (b) methylmethacrylate, producing diallylguanidine (DAG) or its derivatives with acetic and trifluoroacetic acid (DAGAc and DAGTFAc) [2], (meth)acrylateguanidines (AG, MAG) [3,4], methacryloylguanidine (MG) and its derivatives (MGCl, MGAc, MGTFAc) [5,6]. Those new compounds (Scheme 1) possess great potential for tuning both monomers and their (co)polymers.

**Scheme 2:**
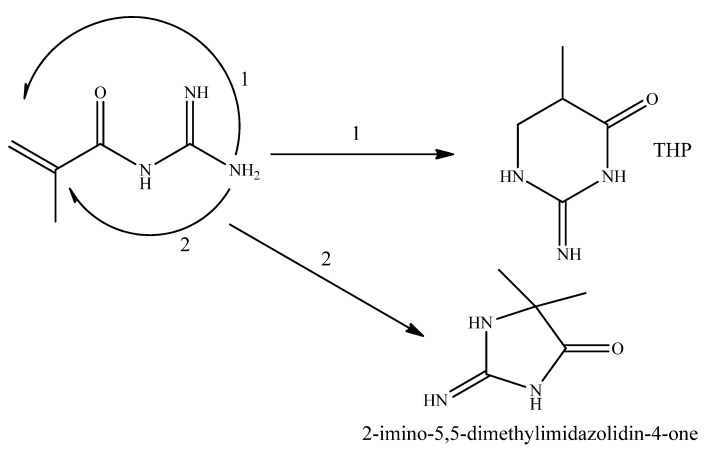
Possible methacryloyl guanidine isomerization paths.

A cyclic byproduct of MG synthesis (Scheme 2) was isolated in [7] and identified as the 2-imino-5-methyltetrahydropyrimidin-4(1H)-one (THP). A general strategy for guanidine-containing monomer synthesis and (co)polymer was employed in this work, allowing for potential synthesis of its methacrylic salt (MTHP) for biocidal polymer development, akin to MAG synthesis [4] and MAG/DADMAC copolymerization [8] described earlier.

Surprisingly, the approach for obtaining water-soluble biocides via radical polymerization of guanidine-containing vinyl monomers has not become as widespread as the classical polycondensation and melt-mixing methods, described, e.g., in [9,10]. This is mostly due to the prevalence of biocide polyhexamethyleneguanidine (PHMG), which is easily synthesized from guanidine salts and hexamethylenediamine (in melt, via microfluidic methods [11], etc.). However, following the 2011 case of ‘fatal misuse’ which has lead to more than 200 directly connected casualties [12] and an exposure rate of about 30% of Korean children [13], PHMG is actively being phased out, regulated, and banned, and a need for different guanidine-based biocides might resurface.

An article from 2016 [14] deals with the copolymerization of vinyl-functionalized oligohexamethyleneguanidine with methylmethacrylate, but the long-term biocidal copolymers obtained are insoluble. A biocidal acrylic copolymer was synthesized by radical polymerization [15], but PHMG was chemically bonded on it afterwards, via epoxy-amine condensation. Side-chain guanidine functionalized 3-guanidinopropyl methacrylamide homopolymerization and its block copolymerization with *N*-(2-hydroxypropyl) methacrylamide via aRAFT was studied in [16], but without a substantial followup. Finally, radical polymerization was employed to graft PHMG onto cellulose fibers [17] for *E. coli* inhibition.

However, most of the work done in the field of radical methacryloylguanidine polymerization with the classical copolymerization approach and are either our from our organization (TIPS RAS) or our colleagues and co-authors (Zaikov, Khashirova, Kabanova, Malkanduev, etc.), with the notable exception of Gorbunova et al. [18], working with acrylate-allylguanidine copolymerization.

## 2. Materials and Methods

DMSO and acetonitrile solvents were purified according to standard practices. Deuterated solvents (DMSO-d_6_ and D_2_O), guanidine hydrochloride (GHC) and ammonium persulfate (PSA) were purchased from Sigma-Aldrich (subsidiary of Merck) and used without additional purification. Methylmethacrylate (MMA), purchased from Merck (Kenilworth, NJ, USA) was distilled in argon flow at 150 mm Hg, temperature range 44–46 °C.

### 2.1. Synthesis of Methacryloylguanidine (MG) and Its Cyclical Analogue 2-Imino-5-methyltetrahydropyrimidin-4(1H)-one (THP)

The synthesis of MG and THP was carried out in either dehydrated acetonitrile or DMSO. Either finely-ground GHC was added to a round flask with an agitator and an addition funnel, then either acetonitrile (0.3 L) solvent followed by MMA (0.052 mL, 0.48 mol) was added dropwise, or DMSO guanidine solution was dropwise added to DMSO MMA solution. A sediment forms on dropwise reagent addition; the solution was agitated for 4 h at room temperature, then in some cases was left overnight. Afterwards, the cyclical product sediment was filtered.

### 2.2. Synthesis of THP Methacrylate (MTHP)

A flat-bottomed 0.25 L flask with a reflux condenser and an agitator was used. Then, 12.7 g (0.1 mol) of THP was added to 0.1 L of water, then agitated, and an equimolar amount of MA (8.6 g) was added. Synthesis was considered complete after the complete dissolution of THP. If deemed necessary, the solution was evaporated on a rotary vaporizer, the residue vacuum-dried to a constant mass (2–3 mm Hg residual pressure). MTHP yield 18.2 g (85%).

### 2.3. MTHP Polymerization

MTHP obtained in previous step was dissolved in distilled water to obtain 0.1 L of 0.4M solution. Radical polymerization was then initiated by 0.12 g PSA at 60 °C. Polymer yield 75% by mass.

### 2.4. MTHP Copolymerization with Diallyldimethylammonium Chloride (DADMAC)

Synthesized MTHP was dissolved in 0.1 L water, then 65% water solution of DADMAC was added to a combined volume of 0.2 L, comonomer ratio 1:1, combined comonomer concentration either 1 or 2 mol/L. 0.228 g PSA was added under constant agitation, then polymerization was carried out at 60 °C for 20 h, followed by sedimentation into acetone. Polymer was additionally purified from monomers by dialysis against distilled water. Copolymer yield was 40 to 51%.

Elemental composition was determined via pyrolysis chromatography on a Thermo Flash 2000 Organic Elemental Analyzer (Thermo Fisher Scientific, Waltham, MA, USA), sample mass 1–4 mg pyrolyzed at 2000 °C in helium carrier gas, to a precision of 3–5%.

Intrinsic viscosity measurements were performed in an Ubbelohde type viscometer (Fungilab, Barcelona, Spain) in water and 0.5 N NaCl solution in water at 30 °C.

Monomer conversion was determined gravimetrically, determined via weighing of the precipitated polymer.

^1^H and ^13^C NMR spectra were collected on a Bruker (Billerica, MA, USA) spectrometers DRX500 (500.13 MHZ for ^1^H and 125.76 MHz for ^13^C), and MDS-300 (300 MHz) in D_2_O and DMSO-d_6_ at 20 °C, chemical shifts relative to solvent residual protons.

## 3. Results and Discussion

### 3.1. THP and MTHP Synthesis

To obtain the goal compound of MTHP, the synthesis of THP itself had to be optimized beforehand.

**Scheme 3:**
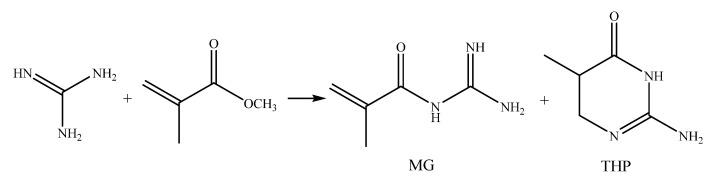
MG and THP synthesis.

THP is a byproduct of MG synthesis from MMA and GHC (Scheme 3), with a yield of about 5–10% [5,6]. Varying the conditions—reagent loading ratio and initial concentrations (0.4 to 4.6 mol/L), reaction medium (dioxane, acetone, MMA, methanol), temperature (20 to 60 °C), does not impact the creation of solid fraction, containing mainly MAG and THP, yielding up to 30%. After decanting the liquid phase contained almost pure goal MG product, obtained by solvent evaporation with a yield of 50–70%.

The order or reagent addition in MG and THP synthesis in DMSO was varied to determine its influence on THP yield [7]: either an ester (MMA or butylmethacrylate, BMA) was added to GHC solution, or vice versa (Table 1). THP yield is increased by slowing the addition of ester, and changing the reagent addition order to ester-first. Dilution of the reaction stock also leads to the shift in product yields to favor THP over MG, so the optimal conditions for THP production were determined to be 0.4 mol GHC in 0.1 or 0.2 L DMSO (Table 1, №2, 4), BMA ester then slow addition of GHC over 40 min and overnight (16+ h) stabilization of reaction stock. THP elemental composition were in accord with the calculated values (Table 2).

### 3.2. Spectral Analysis of MTHP

^1^H NMR of MTHP salts in DMSO-d_6_ was employed to determine their structure, in addition to the synthesized sample of MTHP (Table 3, № 1–3). THP and its salts (№ 4–6), MA (№ 7) and MAG (№ 8) were also added for comparison.

Signals for both methacrylic and pyrimidinone parts of different MTHP are in proximity to each other; comparison of methacrylic signals of MTHP with those of methacrylic acid shows a shift of those for MTHP samples, attributable to the higher pyrimidinone group influence compared to that of an acid proton.

MAG signals are even more strong field, denoting a strong interaction of guanidine part compared to that of a pyrimidinone, due to the counterion volume. N–H proton shifts for MTHP and MAG are similar (7.7–7.8 ppm), due to the similar interaction of methacrylate anion with nitrogen-containing anion, while for the base THP that signal is at 8.35 ppm (№ 4). Ring carbon protons of THP are present in the stronger field of spectrum (cf. № 1–3 with № 4).

Salt formation with strong (compared to MA) HCl and trifluoroacetic acids leads to a weak-field shift of all signals, including N–H (8.65 and 8.79 ppm). All the pyrimidinone signals for THP compounds are present as multiples, with their character varying with the compound particulars, implying complex electron and steric interactions between parts of MTHP derivatives studied.

### 3.3. The Possibility of MTHP (Co)Polymerization

**Scheme 4:**
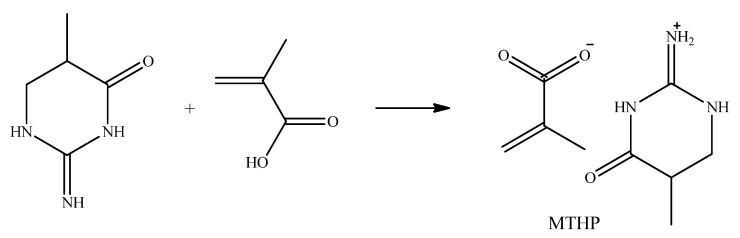
MTHP synthesis.

MTHP (co)polymerization is a way to include the cyclical byproduct in polymerization via creating the salt of THP and MA at an equimolar ratio (Scheme 4), in aqueous media, as all the reagents are water-soluble. Polymerization results are comparable with MAG in [2], yield 60–75%.

MTHP recrystallization with acetone yielded interesting results, as the primary, insoluble in acetone, fraction MTHP-T was determined to be a MA:THP salt with the ratio being 1:3, while the acetone-soluble lesser fraction MTHP-M is a 2:1 MA:THP salt.

Polymerization was carried out directly in solution, after MA and THP equimolar mixing without MTHP separation; the conditions were 0.4 mol/L MTHP, 5 mmol/L PSA and 60 °C, total polymerization time 20 h.

After 30 min the solution grows turbid, and after an hour more, it clears. Post-synthesis analysis of the reaction mixture with an intrinsic viscosity of 1.06 dL/g in water at 30 °C had shown the polymer yield to be about 75% with traces of monomer, demonstrating the polymerization system to be promising.

**Scheme 5:**
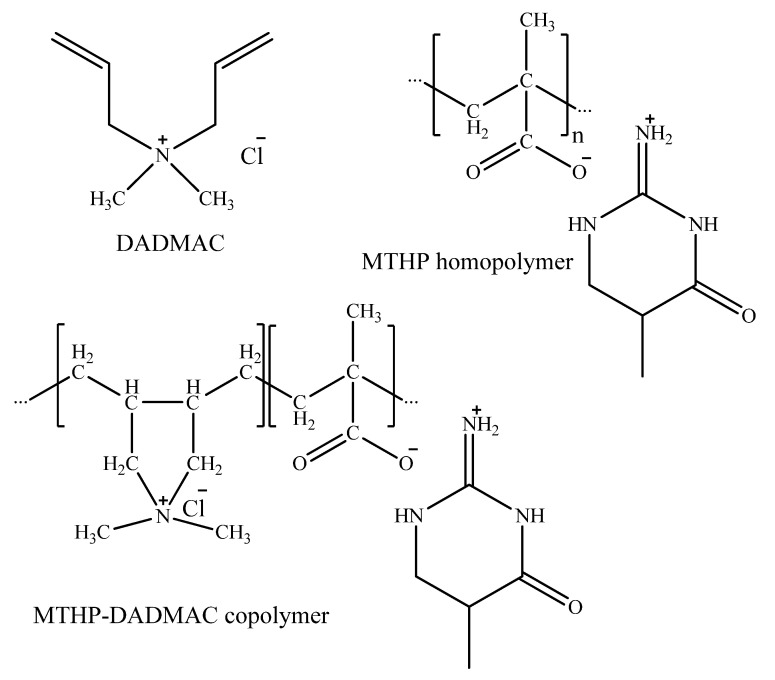
DADMAC and MTHP polymerization products.

Thus, radical MTHP copolymerization with DADMAC (1:1) in same conditions (Table 4, Scheme 5) leads to fairly high yields, with an increase in initial comonomer concentration leading to higher conversion (№ 2,3). Intrinsic viscosity in 0.5N NaCl aqueous solution had shown it to also lead to a viscosity increase (№2—0.11 dL/g, №3—0.14 dL/g), which is in line with basic rules of radical copolymerization. The occurrence of MA units in homopolymer might be attributed to the subtraction of soluble THP hydroxide during dialysis, which requires further investigation.

Preliminary biocidal activity studies demonstrated THP copolymers to be weaker (MIC = 96 μg/mL on *S. aureus*) than similar MAG copolymers (MIC = 32 μg/mL); this might be attributed to the difference in cation size and/or charge distribution [19,20].

## 4. Conclusions

Studying the synthesis and radical (co)polymerization of MTHP had shown it to be a promising platform for production of biocidal (co)polymers at high yields. Further investigation and fine-tuning the experimental conditions of MTHP synthesis and following polymerization is required to further increase the yield and the strength of proposed biocide material.

## Figures and Tables

**Figure 1:**
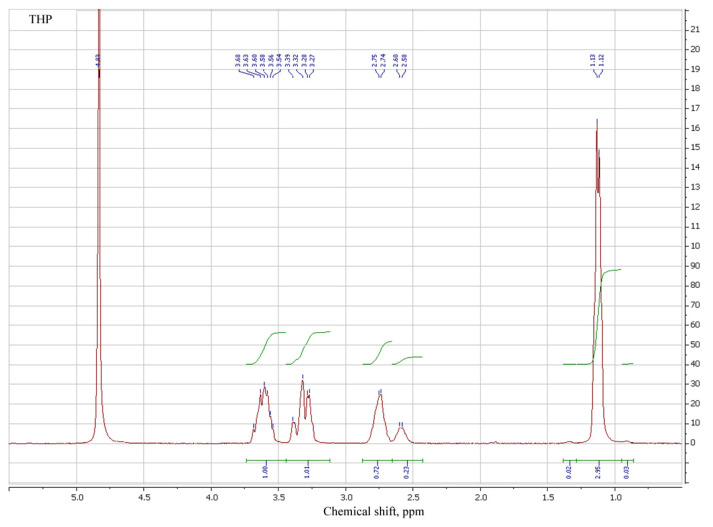
NMR^1^H spectrum of THP in deuterated water.

**Figure 2:**
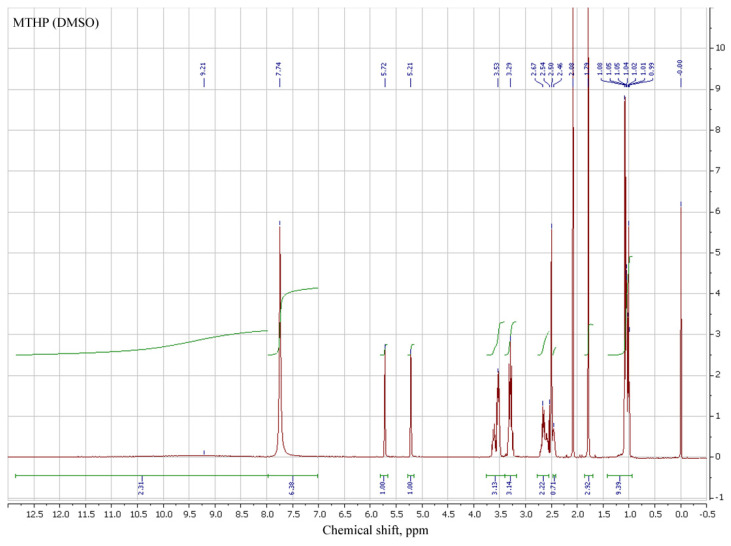
NMR^1^H spectrum of MTHP acetone-insoluble fraction in deuterated DMSO.

**Figure 3:**
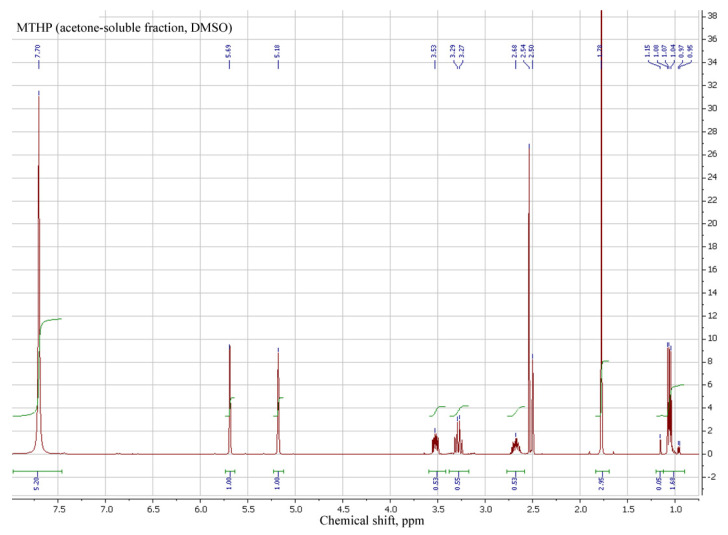
NMR^1^H spectrum of MTHP acetone-soluble fraction in deuterated DMSO.

**Figure 4:**
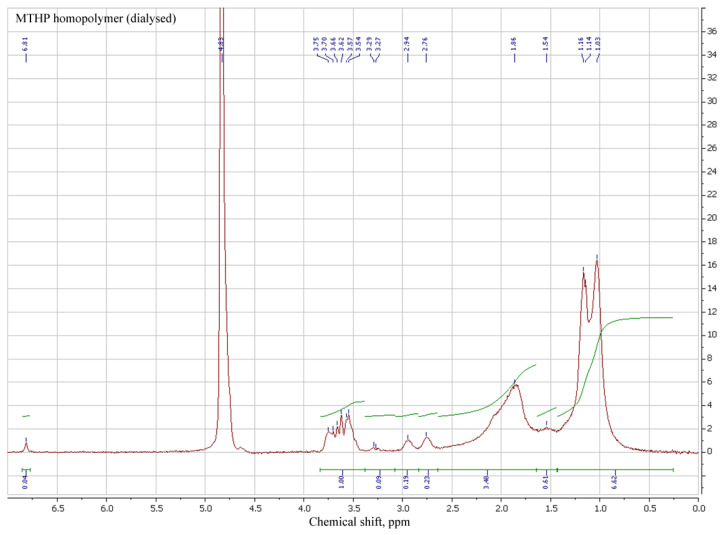
NMR^1^H spectrum of MTHP homopolymer in deuterated water after dialysis.

**Figure 5:**
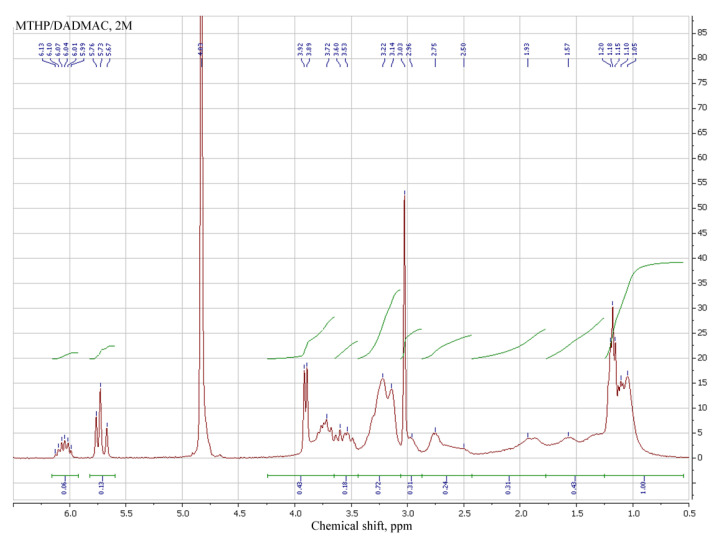
NMR^1^H spectrum of MTHP/DADMAC copolymer in deuterated water (soluble fraction after dialysis, [M]_Σ2_ = 2M).

**Figure 6:**
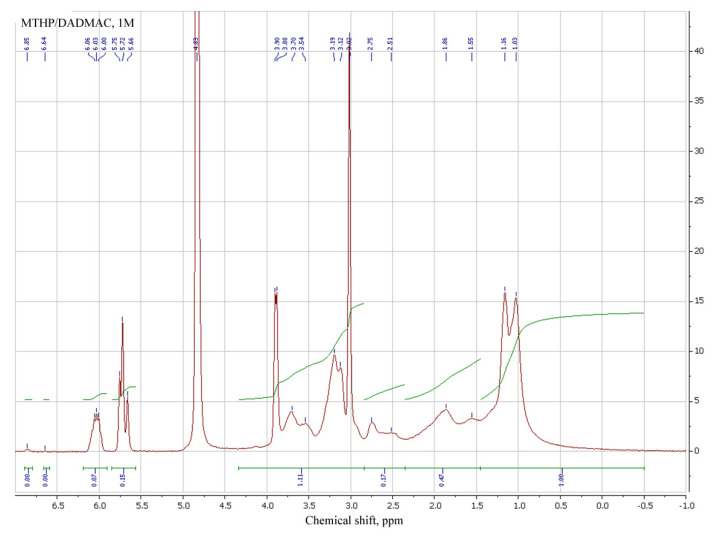
NMR^1^H spectrum of MTHP/DADMAC copolymer in deuterated water (insoluble fraction after dialysis, [M]_Σ1_ = 1M).

**Table 1:** Conditions for MG synthesis in DMSO.

№	Reagent Order	Ester	Time, min		Yield		
			Dropwise Adding	Synthesis	MG		THP
					g (mol)	Mol, %	Mol, %
1	GHC, then ester	MMA	30	240	27.1 (0.2131)	53	31
2		BMA	40	240+ overnight	0.15 (0.0017)	3	92
3	Ester, then GHC	MMA	45	240	15.4 (0.1212)	30	62
4		BMA	130	240+ overnight	4.9 (0.0463)	12	87

**Table 2:** THP elemental composition under varied synthesis conditions.

№	Elements, Mass.%			
	C	H	N	N/C
Calculated	47.23	7.14	33.05	0.6998
1	47.87	7.10	33.04	0.6902
2	47.75	7.16	32.86	0.6882
3	47.20	7.11	33.19	0.7032

**Table 3:** ^1^H NMR spectral characteristics in DMSO-d_6_ of MTPH, TPH, its salts, methacrylic acid (MA) and MAG ^1^ (Figure 1, Figure 2 and Figure 3).

№	Sample	Chemical Shift, ppm						NH, δ
		Methacrylic Part, δ			Pyrimidinone Part, δ			
		CH_3_	CH_A_	CH_B_	CH_3_	CH	CH_2_	
1	MTHP	1.78	5.21	5.71	1.06	~2.4 + 2.67	3.27 + 3.50	7.74
2	MTHP-T	1.79	5.21	5.72	1.05	2.46 + 2.67	3.29 + 3.53	7.74
3	MTHP-M	1.78	5.18	5.69	1.07	2.68	3.28 + 3.53	7.70
4	THP		—	—	1.01	2.32 + 2.75	3.00 + 3.35	8.35
5	THP-TFAc		—	—	1.08	2.83	3.24 + 3.55	8.65
6	THP-HCl		—	—	1.06 + 1.13	2.84 + 3.07	3.22 + 3.55 + 3.69	8.79
7	MA	1.83	5.59	5.87		—	—	—
8	MAG	1.76	5.05	5.60		—	—	7.83

^1^ δ—chemical shift, ppm; all the pyrimidinone signals are multiplets.

**Table 4:** Composition and yield of MTHP (co)polymerization products with MA and DADMAC (water, 60 °C, PSA 5 mmol/L, 20 h) ^1^ (Figure 4, Figure 5 and Figure 6).

№	THP, mol/L	MA, mol/L	DADMAC, mol/L	(Co)Polymer Composition				K, %
				NMR		Elemental Analysis		
				M_1_	M_2_	M_1_	M_2_	
1	0.4	0.4	—	38	62	41	59	53
2	0.5	0.5	0.5	51	48	45	55	41
3	1.0	1.0	1.0	67	33	77	23	51

^1^ K—conversion of monomers to polymers, M_1_—MTHP, M_2_—DADMAC (or MA in № 1).

## Data Availability

The data presented in this study are available on request from the corresponding author.
